# Progress in quickly finding orthologs as reciprocal best hits: comparing blast, last, diamond and MMseqs2

**DOI:** 10.1186/s12864-020-07132-6

**Published:** 2020-10-24

**Authors:** Julie E. Hernández-Salmerón, Gabriel Moreno-Hagelsieb

**Affiliations:** Wilfrid Laurier University, Department of Biology, 75 University Ave W, Waterloo, N2L 3C5 ON Canada

**Keywords:** Orthologs, Reciprocal best hits, Fast algorithms, Sequence comparison

## Abstract

**Background:**

Finding orthologs remains an important bottleneck in comparative genomics analyses. While the authors of software for the quick comparison of protein sequences evaluate the speed of their software and compare their results against the most usual software for the task, it is not common for them to evaluate their software for more particular uses, such as finding orthologs as reciprocal best hits (RBH). Here we compared RBH results obtained using software that runs faster than blastp. Namely, lastal, diamond, and MMseqs2.

**Results:**

We found that lastal required the least time to produce results. However, it yielded fewer results than any other program when comparing the proteins encoded by evolutionarily distant genomes. The program producing the most similar number of RBH to blastp was diamond ran with the “ultra-sensitive” option. However, this option was diamond’s slowest, with the “very-sensitive” option offering the best balance between speed and RBH results. The speeding up of the programs was much more evident when dealing with eukaryotic genomes, which code for more numerous proteins. For example, lastal took a median of approx. 1.5% of the blastp time to run with bacterial proteomes and 0.6% with eukaryotic ones, while diamond with the very-sensitive option took 7.4% and 5.2%, respectively. Though estimated error rates were very similar among the RBH obtained with all programs, RBH obtained with MMseqs2 had the lowest error rates among the programs tested.

**Conclusions:**

The fast algorithms for pairwise protein comparison produced results very similar to blast in a fraction of the time, with diamond offering the best compromise in speed, sensitivity and quality, as long as a sensitivity option, other than the default, was chosen.

## Background

Finding orthologs is an important step in comparative genomics and represents a central concept in evolution. Orthologs are defined as characters that diverge after a speciation event [1]. This normally means that, if the characters are genes, then they can be thought of as the same genes in different species. Because of their relationship, orthologs are expected to typically conserve their original function, an inference that has been supported by several lines of evidence [2–5].

Efforts in standardizing methods for the inference of orthology remain in constant evaluation, with over forty web services available to the community [6, 7]. Few of these methods are based on phylogenetic analyses (tree-based approach), which, despite expected to be the most accurate, tend to be computationally intensive and impractical for big databases [8, 9]. Some methods employ pairwise sequence similarity comparisons (graph-based methods) that have been successfully implemented, such as the clusters of orthologous groups (COG) database [10, 11]. However, researchers have an increasing need to produce their own sets of orthologs, as genome sequencing has become a much more commonly available technology.

Perhaps the most common approach, or operational definition, of orthology, is Reciprocal Best Hits (RBH), which is a simple method that has shown low false-positive rates and ease of implementation [9, 12, 13]. Essentially, the complete set of proteins encoded by the annotated genes of a genome, its proteome, is compared to other proteomes. If two proteins, each encoded in a different genome, find each other as the best/highest-scoring matches among the proteome of the opposite genome, they are RBH and thus inferred to be orthologs. The most common program for comparing proteomes is blastp [14]. This program was chosen for being the fastest available at the time when comparative genomics began (*v.g.* [15, 16]). However, the amount of sequences to analyze continues to grow exponentially, making the speed of blastp comparisons too slow for the increasing demand for sequence analysis.

When authors introduce a software suite for sequence comparison, they often compare the speed and overall performance of their software to blastp. Since speed tends to come at a cost in sensitivity and accuracy, the reports might include differences in performance in overall sequence comparison and number of detected sequences. However, more specialized usages, like finding orthologs as RBH, which do not often require the finding of every sequence that would be found by blastp, might be affected differently. Thus, it becomes necessary to test the adequacy of the software in particular tasks. Accordingly, prior work compared the performance of three fast programs against blastp [17]. The programs tested were blat [18], ublast [19] and lastal [20], with lastal producing the most-similar-to-blastp results. Since then, two more recent programs for fast sequence comparison have been developed: diamond [21] and MMseqs2 [22] (from now on mmseqs). Here we use lastal as a reference to the previous report [17] and test the performance of these two new programs, diamond and mmseqs, for obtaining RBH.

## Results

### Runtimes

**Fig. 1:**
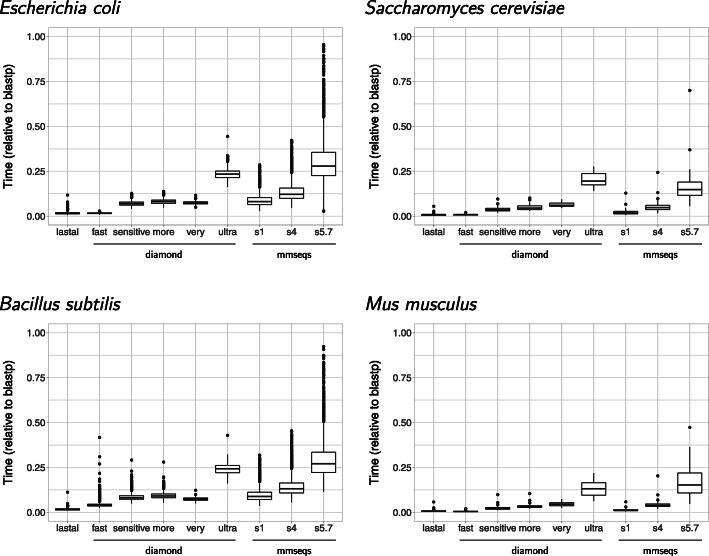
Difference in speed obtaining pairwise alignments. The times plotted are the “real” times, as measured by the *time* UNIX command, relative to blastp. The fastest of the three programs we tested was lastal. Both diamond and mmseqs were tested with different sensitivity options

The computing speeds for finding homologs were plotted for each program relative to blastp. Of all the programs tested, lastal was the fastest (Fig. 1), obtaining results in a median of approximately 1.5% of the blastp time to run with bacterial proteomes (Fig. 1, left) and 0.6% with eukaryotic ones (Fig. 1, right). The proportion of time saved running any of the fast programs was much more evident when running comparisons between eukaryotic proteomes, which contain larger numbers of proteins than bacterial ones.

Both diamond and mmseqs offer different sensitivity options. The sensitivity modes offered by diamond are “fast”, “sensitive”, “more-sensitive”, “very-sensitive” and “ultra-sensitive”. The fast mode was the closest in runtime to lastal. The other options took increasingly longer to run, mostly according to their level of sensitivity (Fig. 1, left).

The sensitivity options for mmseqs tested were 1, 4, and 5.7. 1 and 4 were chosen because both were used in the article presenting the software [22], while 5.7 is the default option. As expected, the 5.7 option saved the least time in most cases, except in *S. cerevisiae*, where diamond with the ultra-sensitive setting was slower (Fig. 1, top-right).

### Reciprocal best hits

**Fig. 2:**
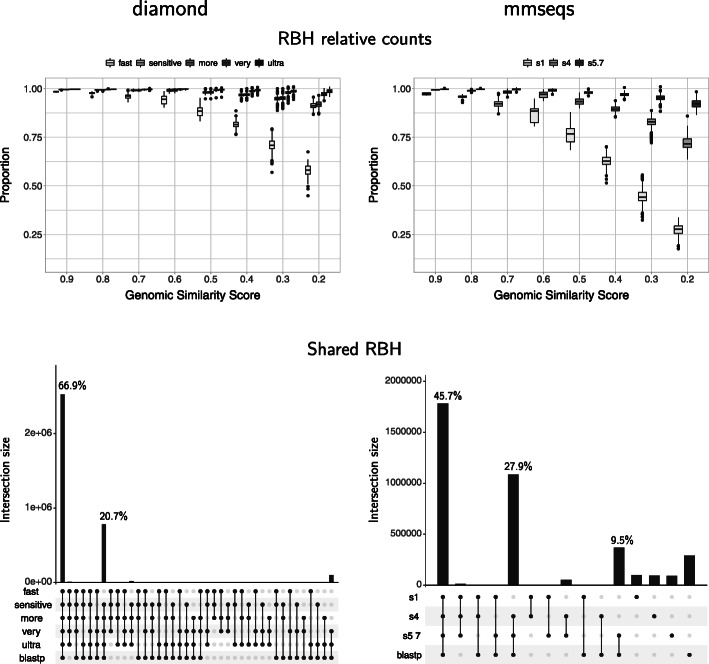
Reciprocal best hits found by diamond and mmseqs. The figure shows results using the *E. coli* proteome as reference. Results with the other reference proteomes showed similar tendencies (see supplementary document). The proportion of RBH found is comparable to those found by blastp when the proteomes involved are very similar (high Genomic Similarity Scores, *GSS*). This proportion reduces with the *GSS*. As expected, low sensitivities reduced the proportion of RBH found. Note that the improvement between diamond’s very-sensitive and ultra-sensitive settings is modest compared to their relative runtimes (Fig. 1)

**Fig. 3:**
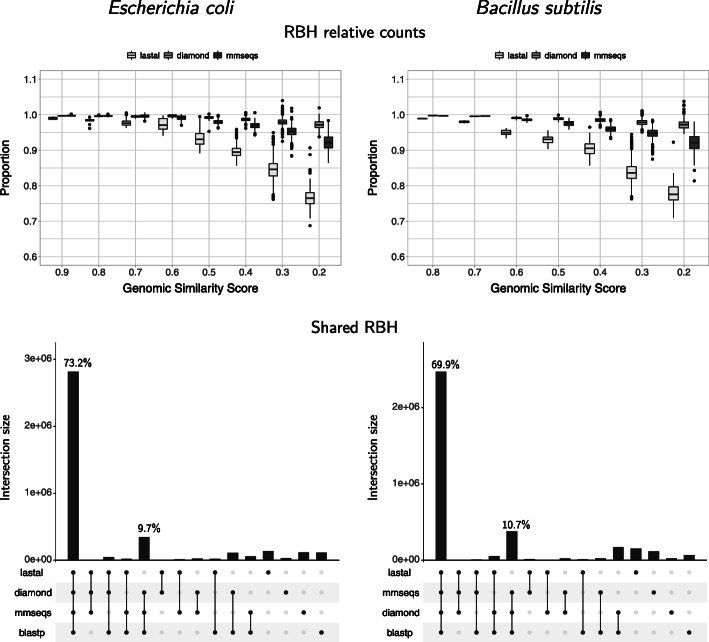
Reciprocal best hits found by all programs with bacterial proteomes. The proportion of RBH obtained with the fast programs is very similar to those obtained with blastp when genomes are very similar to each other (high Genomic Similarity Scores, *GSS*). As *GSS* decreases, the proportion falls. Both diamond and mmseqs showed improved proportions at low *GSS* compared to lastal. Close to 70% of the RBH detected are shared by all programs. Both diamond and mmseqs share the most RBH with blastp

**Fig. 4:**
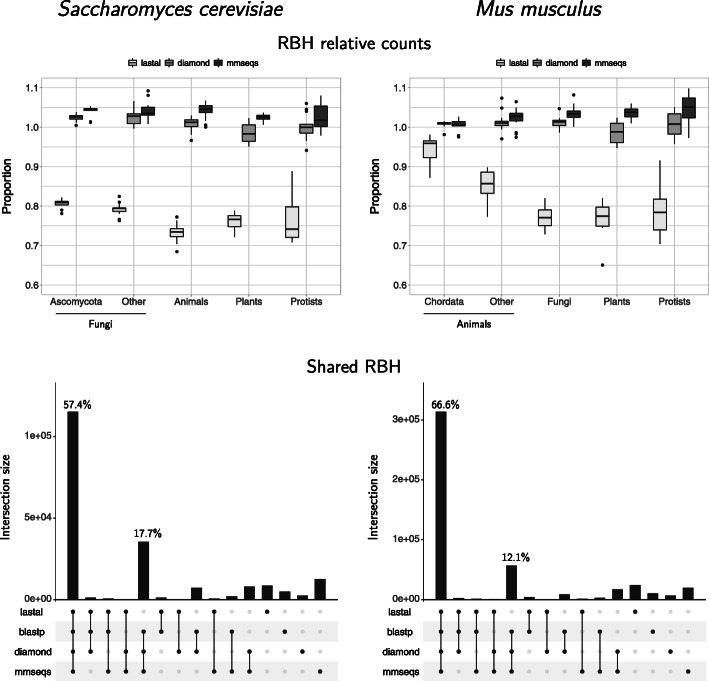
Reciprocal best hits found by all programs with eukaryotic proteomes. The proportion of RBH obtained with the fast programs, relative to the number obtained with blastp, is more similar when genomes are in the same taxonomic group as the reference genome (Ascomycota for *S. cerevisiae*, Chordata for *M. musculus*). Both diamond and mmseqs showed improved proportions compared to lastal. Bottom: most of the RBH detected are shared by all programs. Both diamond and mmseqs share the most RBH with blastp

The proportion of reciprocal best hits found using the fast programs was also evaluated with respect to blastp (Figs. 2, 3 and 4, Supplementary Figures S1–S3). Our results showed a very similar proportion of RBH to those obtained with blastp when the compared proteomes were more similar to each other (higher values of genomic similarity or *GSS*). In all cases, as the *GSS* decreased, so did the proportion of RBH found.

#### Sensitivity options

The different sensitivity options resulted in different proportions of RBH found by either diamond or mmseqs (Fig. 2, Supplementary Figures S1–S3). The differences in results become more obvious as the genomic similarity drops. The results below refer to the figures obtained with the *E. coli* reference genome (Fig. 2), though the results with other reference genomes were similar (Supplementary Figures S1–S3).

The proportions of RBH found using diamond’s fast setting, which is the program’s default, dropped noticeably compared to those detected with the other options (Fig. 2, top-left). The rest of the options found increasing proportions of RBH in accordance to the level of sensitivity, albeit with small differences. All options other than the default found more than 0.90 of the results found by blastp at the lowest end of genomic similarity, with the very-sensitive and ultra-sensitive modes finding more than 0.96 of the RBH found by blastp.

The UpSet plot showed that the sensitive to ultra-sensitive settings had the highest RBH in common with blastp for a total of 87.6% (66.9*%*+20.7*%*) (Fig. 2, bottom-left).

In the case of mmseqs, the sensitivity options tested produced noticeably different results (Fig. 2, right). Again, the top options, 4 and 5.7, shared the most results with blastp (Fig. 2), though only amounting to a combined 73.6% (45.7*%*+27.9*%*). The 5.7 option produced the best results, with 9.5% more RBH shared with blastp.

#### Programs

Both diamond with the very-sensitive option and mmseqs ran with the 5.7 setting detected a higher proportion of RBH than lastal (Figs. 3 and 4). This was true even at the lowest *GSS* values, meaning that even in the worst cases, neither diamond, nor mmseqs, would miss more than 10% of the RBH that would be produced by blastp. The diamond results were the best in this sense.

With bacterial proteomes, close to 70% of all RBH were detected by all programs (Fig. 3, bottom). The second most important intersections, for both *E. coli* and *B. subtilis*, shows that diamond and mmseqs shared the majority of RBH with blastp (making up a total of 73.2+9.7=82.9*%* and 69.9+10.7=80.6*%*, respectively). Unlike our previous analysis [17], which showed evidently higher percentages of RBH detected solely by blastp, the proportion of RBHs exclusive to each program were somewhat similar. These exclusive RBH seem to represent differences in sensitivity, which might correspond to a mixture of differentially detected true and false positives.

In contrast to the results in bacteria, both diamond and mmseqs produced a higher proportion of RBH than blastp in eukaryotes (Fig. 4, top). The proportion of RBH found by mmseqs was the highest.

The UpSet plots showed a lower proportion of shared RBH in comparisons involving eukaryotic proteomes than in those involving prokaryotic ones. The intersection of all programs covered close to 62% of the RBH detected (Fig. 4, bottom). Again, diamond and mmseqs shared the most RBH with blastp (57.4+17.7=75.1*%* in *S. cerevisiae* and 66.6+12.1=78.7*%* in *M. musculus*).

### Error estimates

**Fig. 5:**
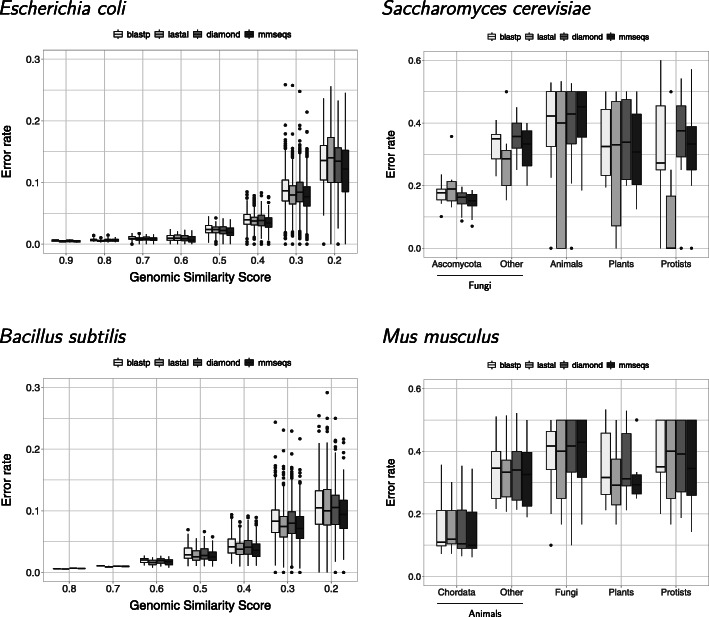
Error rate estimates. All error rate estimates are very close between programs, indicating that using any of the fast programs would not add errors beyond what would be obtained with blastp. As expected, error rate estimates increase with genome divergence. The error rate estimates are higher in eukaryotes (note that the scale is different to that for bacteria), presumably due to the complex dynamics of eukaryotic chromosomes

Error rates increased with proteome divergence (Fig. 5). The error rates were very similar among all programs. The mmseqs results consistently showed the lowest error rate estimates. Overall, eukaryotes error rates were higher than those estimated for prokaryotes (Fig. 5, right).

## Discussion

### The fastest program was lastal

The highest sensitivity options offered by diamond, very-sensitive and ultra-sensitive, were introduced with version 2.0.0 of the program, which was released while this report was under review. Thus, this might be the first article showing results using them. As mentioned in the “Results” section, the fast mode, which is the default, was the closest in runtime to lastal. The other options took increasingly longer to run. However, with bacterial proteomes the very-sensitive mode ran in a time between that taken by the fast and sensitive modes (Fig. 1, left). The ultra-sensitive mode was the slowest to run, breaking the “staircase-step” length by a large gap (Fig. 1, overall).

With mmseqs, the 5.7 option saved the least time in most cases, except in *S. cerevisiae*, where diamond with the ultra-sensitive setting was slower (Fig. 1, top-right).

Note that we ran mmseqs with the “easy-search” function. This function produces any output format desired without much user intervention. The easy-search function accepts the target either in plain fasta format, or as a formatted database, but the query has to remain in plain fasta format. Another way to produce pairwise alignments with mmseqs would use the “search” function instead of easy-search. The search function requires databases built for both, query and target. The results of the search function is also in database format. This database can then be used to extract results into other output formats as necessary.

Also note that the mmseqs software can also precompute indexes for its databases. We decided not to build indexes because they take very long to be built and use too much space. For example, the database for the largest bacterial proteome (12,103 annotated proteins) used 5.7M of space, which increased to 898M when building the index. Runtimes might vary if the user preferred to build a database index and use the search function instead of the easy-search one.

Finally, note that mmseqs has a “rbh” function, with a future version offering an “easy-rbh” function, which should take care of producing a table without much user intervention (Martin Steinegger, personal communication). However, we decided not to use the “rbh” function because we preferred to keep control of the parameters used to produce RBH.

### The best compromise for obtaining reciprocal best hits was diamond with the very-sensitive option

As mentioned under results, both diamond and mmseqs were run with different sensitivity options, which, as expected, resulted in different proportions of RBH found (Fig. 2, Supplementary Figures S1–S3). At the lower end of genomic similarity, the differences in results among the tested sensitivity options became more apparent. The results discussed below refer to those obtained with the *E. coli* reference genome (Fig. 2). However, the results with other reference genomes showed similar tendencies (Supplementary Figures S1–S3).

In the case of diamond, the lower proportions found using the fast setting, the program’s default, was apparent even when comparing very similar proteomes (Fig. 2, top-left). The rest of the options behaved noticeably better, which suggests that diamond with the sensitive mode would already be a good substitute for blastp. The increase in RBH between the sensitive and the more-sensitive options was small, with a somewhat larger gap between the more-sensitive and the very-sensitive modes and, finally, another slight increase from the very-sensitive to the ultra-sensitive mode. These tendencies are more obvious at the lowest *GSS*, where the proportion of RBH found by diamond with the very-sensitive and ultra-sensitive modes were above 0.96 (Fig. 2, top-left).

The UpSet plots showed that the sensitive to ultra-sensitive settings had the highest RBH in common with blastp for a total of 87.6% (66.9% + 20.7%) (Fig. 2, bottom-left), with 20.7% representing the difference in results compared to the fast option. Thus, diamond with the fast option would perform very poorly compared to blastp. It also appears that the ultra-sensitive mode had very little advantage over the very-sensitive option, considering the much longer time it took to run (Fig. 1). This setting took a median of 7.4% of the blastp time to run with bacterial proteomes (Fig. 1, left) and 5.2% with eukaryotic ones (Fig. 1, right). These results are the reason why we selected this option to represent diamond in the overall software comparison.

The top options tested for mmseqs, 4 and 5.7, shared the most results with blastp (Fig. 2), with The 5.7 option producing the best results, with 9.5% more RBH shared with blastp than the other options. We thus chose the 5.7 setting, which is the program’s default, for comparisons against results obtained with the other fast programs.

At the sensitivity levels selected above, both diamond and mmseqs detected a higher proportion of RBH than lastal (Figs. 3 and 4). This was true even at the lowest *GSS* values, meaning that even in the worst cases, neither diamond, nor mmseqs, would miss more than 10% of the RBH produced by blastp. The diamond results were the best in this regard.

With bacterial proteomes, close to 70% of all RBH were detected by all programs (Fig. 3, bottom). The second most important intersections, for both *E. coli* and *B. subtilis*, shows that diamond and mmseqs shared the majority of RBH with blastp (see “Results” section). Unlike our previous analysis [17], which showed evidently higher percentages of RBH detected solely by blastp, the proportion of RBHs exclusive to each program were somewhat similar. These exclusive RBH seem to represent differences in sensitivity, which might correspond to a mixture of differentially detected true and false positives.

In contrast to what we observed in bacteria, both diamond and mmseqs produced a higher proportion of RBH than blastp in eukaryotes (Fig. 4, top). The proportion of RBH found by mmseqs was the highest. Since these proportions are above the RBH found by blastp, it is difficult to decide if these results are an improvement or a problem. The error rate estimates did not help deciding (see section below and Fig. 5, right).

The results of all programs shared a lower proportion of RBH in the eukaryotic results than in the prokaryotic ones, with the intersection of all programs covering close to 62% of the RBH detected (Fig. 4, bottom).

### Error rate estimates were very similar among all programs

Despite genomic rearrangements and horizontal gene transfer result in divergence of gene order, a few regions are preserved even between the genomes of evolutionarily distant organisms [23–25]. Thus, despite conservation of adjacency is a very limited source for correction of misidentified orthologs, pairs of adjacently conserved genes can still be used to estimate error rates [12].

As expected, error rates increased with proteome divergence (Fig. 5). The error rates were very similar among all programs. The mmseqs results consistently showed the lowest error rate estimates. These results suggest that the quality of orthologs remains as high, if not better, when using software that produces results faster than blastp. The reason why mmseqs showed the best quality could be that this program uses a very efficient implementation of the Smith-Waterman algorithm to produce its final alignments.

The contrast between bacterial and eukaryotic RBH results might due to the complex dynamics of eukaryotic chromosomes, resulting in complex homology relationships (e.g. [26, 27]). Such complexity might result in differences in paralog/ortholog resolutions. Problems resolving ortholog/paralog relationships might also result in differences in error rates. Accordingly, the error rate estimates were higher for eukaryotes (Fig. 5, right). Besides difficulties for such a simple method as RBH for solving ortholog/paralog relationships, the simple concepts of orthology and paralogy might not be easily applicable to complex situations, where gene conversions, duplications, and loses, complicate the picture [26, 27]. Thus, though we expected to find higher error estimates in eukaryotes, these estimates might reflect both, authentic mistakes, as well as the complexity of eukaryotic genome dynamics.

## Conclusions

The results above suggest that diamond, ran with the very-sensitive option, might be the best alternative to obtain RBH in terms of speed, sensitivity and quality. Our results also showed that the faster programs produced results with very similar error rate estimates as blastp. Improvements in speed were much more evident for the large databases involving eukaryotic proteomes.

## Methods

For these tests we used the sets of annotated protein sequences, or proteomes, from four reference genomes: the bacteria *Escherichia coli* K-12 MG1655 (RefSeq assembly id: GCF_000005845), and *Bacillus subtilis* 168 (GCF_000009045); as well as the eukaryotes *Saccharomyces cerevisiae* S288C (GCF_000146045) and *Mus musculus* (GCF_000001635).

To compare against bacterial references, we used the annotated protein sequences of 3,312 non-redundant prokaryotic genomes. These non-redundant representatives were selected from approximately 16,000 complete prokaryotic genomes available at NCBI’s refSeq genome database [28] by January 2020. To select these genomes, we clustered them using a trinucleotide DNA signature [29], with a *δ*-distance cutoff of 0.04 as described previously [30], resulting in 3,312 clusters. A distance that roughly corresponds to a “species” level. We took one genome per cluster, selecting the genome with the largest number of annotated proteins.

For the comparisons involving eukaryotes, we chose 78 genomes, each selected to represent the members of each eukaryotic taxonomic class with genomes available at the RefSeq database by July 2020. Since eukaryotic genomes often have more than one protein annotated per gene, mostly to account for alternative splicing, we cleaned up the eukaryotic proteomes by writing an ad hoc program to leave only one protein per gene. This way, for example, the mouse, *Mus musculus*, proteome was reduced from 84,985 to 21,905 representative proteins.

Four programs were used to perform protein sequence comparisons: (1) blastp version 2.10.0+ [14], (2) lastal version 1045 [20], (3) diamond version 2.0.2 [21], and (4) mmseqs version 11-e1a1c [22].

To compare times, each pairwise comparisons was run in the same computer, with no other programs running at the same time. Times were obtained by using the unix “time” command. This command reports user, cpu and real times. The plotted times were the real times. The computer was a six-core 2019 Mac mini with 64 GB of RAM. All programs were run to use four of the six available cpu threads in the machine.

To find Reciprocal Best Hits (RBH), we wrote a wrap-up program, getRBH.pl [31], to standardize the options and outputs from the different sequence comparison programs. The options followed the work previously described by Ward and Moreno-Hagelsieb [17]. Namely, the e-value threshold was 1×10^−6^ (1e-6), coverage of 60% of the shortest protein in the alignment, as well as soft-masking and Smith-Waterman alignment, when available (diamond does not have soft masking, thus it was run with no masking). The latter two options were previously found to improve the finding and quality of RBH with blastp [12].

All four programs can work with their own target databases (also called subject databases). These databases make the sequences easier to access and analyze, for example, by reducing the sequence alphabet, indexing for quick retrieval, sometimes precomputing “seeds” (sequence fragments used for a quick selection of sequences that might produce good scores when fully aligned), or any other formatting for the efficient use of the respective sequence comparison programs. These databases are built using a program within the suite: “makeblastdb” for blastp, “lastdb” for lastal; or by a command within the program: “diamond makedb” for diamond, “mmseqs createdb” for mmseqs. We built databases for all programs (automatically implemented in our getRBH.pl wrap-up).

Genomic Similarity Scores (*GSS*) were calculated from blastp results as the sum of the bit scores of all reciprocal best hits *(compScore)* divided by the bit scores of the respective orthologs against themselves *(selfScore)*. This calculation corresponds to the *GSSa* described in [30].

The estimate of error rates relied on conservation of gene order. Ideally, if two adjacent genes, *a* and *b* are homologs each to two corresponding genes *a*^′^ and *b*^′^ in a different genome, then if one of the pairs *a*−*a*^′^ or *b*−*b*^′^ consists of orthologs, then the other pair should also consist of orthologs [12]. In such cases, both pairs are counted as correct inferences. If the program finds instead that the other pair consists of paralogs, the paralog pair is counted as a mistake. The error estimate is thus: *E*=*P*/(*P*+*O*), where *P* is the number of paralog pairs found where an orthologous one was expected [12].

To show the intersection sizes between compared RBH datasets, in lieu of Venn/Euler diagrams, which are hard to draw and interpret when more than three sets are involved, we built matrix-based layouts using UpSetR v1.4.0 [32] for R v. 4.0.2 [33]. Other graphs were also drawn with R.

## Supplementary information

**Additional file 1** Supplementary Figures.

## Data Availability

A wrap-up program for obtaining reciprocal best hits with the software and options tested is available at github: https://github.com/Computational-conSequences/SequenceTools.

## References

[1] Fitch WM (2000). Homology a personal view on some of the problems. Trends Genet.

[2] Chen X, Zhang J (2012). The ortholog conjecture is untestable by the current gene ontology but is supported by RNA sequencing data. PLoS Comput Biol.

[3] Altenhoff AM, Studer RA, Robinson-Rechavi M, Dessimoz C (2012). Resolving the ortholog conjecture: orthologs tend to be weakly, but significantly, more similar in function than paralogs. PLoS Comput Biol.

[4] Gabaldón T, Koonin EV (2013). Functional and evolutionary implications of gene orthology. Nat Rev Genet.

[5] Escorcia-Rodríguez JM, Esposito M, Freyre-González JA, Moreno-Hagelsieb G. Non-synonymous to synonymous substitutions suggest that orthologs tend to keep their functions, while paralogs are a source of functional novelty. bioRxiv. 2020;12. 10.1101/354704. 10.7717/peerj.13843 PMC9440661 36065404

[6] Dessimoz C, Gabaldón T, Roos DS, Sonnhammer ELL, Herrero J, the Quest for Orthologs Consortium (2012). Toward community standards in the quest for orthologs. Bioinformatics.

[7] Boeckmann B, Capella-Gutierrez S, Dalquen DA, DeLuca T, Huerta-Cepas J, Linard B, Pereira C, da Silva AS, Train C-M, Bork P, Lecompte O, von Mering C, Sjölander K, Jensen LJ, Altenhoff AM, Gabaldón T, Thomas PD, Forslund K, Sonnhammer E, Pryszcz LP, Schreiber F, Szklarczyk D, Xenarios I, Martin MJ, Muffato M, Lewis SE, Dessimoz C, Quest for Orthologs consortium (2016). Standardized benchmarking in the quest for orthologs. Nat Methods.

[8] Kuzniar A, van Ham RCHJ, Pongor S, Leunissen JAM (2008). The quest for orthologs: finding the corresponding gene across genomes. Trends Genet.

[9] Kristensen DM, Wolf YI, Mushegian AR, Koonin EV (2011). Computational methods for Gene Orthology inference. Brief Bioinform.

[10] Tatusov RL, Galperin MY, Natale DA, Koonin EV (2000). The COG database: a tool for genome-scale analysis of protein functions and evolution. Nucleic Acids Res.

[11] Galperin MY, Kristensen DM, Makarova KS, Wolf YI, Koonin EV (2017). Microbiala genome analysis: the COG approach. Brief Bioinform.

[12] Moreno-Hagelsieb G, Latimer K (2008). Choosing BLAST options for better detection of orthologs as reciprocal best hits. Bioinformatics.

[13] Wolf YI, Koonin EV (2012). A tight link between orthologs and bidirectional best hits in bacterial and archaeal genomes. Genome Biol Evol.

[14] Camacho C, Coulouris G, Avagyan V, Ma N, Papadopoulos J, Bealer K, Madden TL (2009). BLAST+: architecture and applications. BMC Bioinformatics.

[15] Tatusov RL, Koonin EV, Lipman DJ (1997). A genomic perspective on protein families. Science.

[16] Huynen MA, Bork P (1998). Measuring genome evolution. Proc Natl Acad Sci USA.

[17] Ward N, Moreno-Hagelsieb G (2014). Quickly finding orthologs as reciprocal best hits with BLAT, LAST, and UBLAST: How much do we miss?. PLoS ONE.

[18] Kent WJ (2002). BLAT–the BLAST-like alignment tool. Genome Res.

[19] Edgar RC (2010). Search and clustering orders of magnitude faster than BLAST. Bioinformatics.

[20] Kiełbasa SM, Wan R, Sato K, Horton P, Frith MC (2011). Adaptive seeds tame genomic sequence comparison. Genome Res.

[21] Buchfink B, Xie C, Huson DH (2015). Fast and sensitive protein alignment using DIAMOND. Nat Methods.

[22] Steinegger M, Söding J (2017). MMseqs2 enables sensitive protein sequence searching for the analysis of massive data sets. Nat Biotechnol.

[23] Dandekar T, Snel B, Huynen M, Bork P (1998). Conservation of gene order: a fingerprint of proteins that physically interact. Trends Biochem Sci.

[24] Tamames J (2001). Evolution of gene order conservation in prokaryotes. Genome Biol.

[25] Moreno-Hagelsieb G, Treviño V, Pérez-Rueda E, Smith TF, Collado-Vides J (2001). Transcription unit conservation in the three domains of life: a perspective from Escherichia coli. Trends Genet.

[26] Gogarten JP, Olendzenski L (1999). Orthologs, paralogs and genome comparisons. Curr Opin Genet Dev.

[27] Forslund K, Pereira C, Capella-Gutierrez S, da Silva AS, Altenhoff A, Huerta-Cepas J, Muffato M, Patricio M, Vandepoele K, Ebersberger I, Blake J, Fernández Breis JT, Boeckmann B, Gabaldón T, Sonnhammer E, Dessimoz C, Lewis S, Quest for Orthologs consortium (2018). Gearing up to handle the mosaic nature of life in the quest for orthologs. Bioinformatics.

[28] Haft DH, DiCuccio M, Badretdin A, Brover V, Chetvernin V, O’Neill K, Li W, Chitsaz F, Derbyshire MK, Gonzales NR, Gwadz M, Lu F, Marchler GH, Song JS, Thanki N, Yamashita RA, Zheng C, Thibaud-Nissen F, Geer LY, Marchler-Bauer A, Pruitt KD (2018). RefSeq: an update on prokaryotic genome annotation and curation. Nucleic Acids Res.

[29] Campbell A, Mrázek J, Karlin S (1999). Genome signature comparisons among prokaryote, plasmid, and mitochondrial DNA. Proc Natl Acad Sci USA.

[30] Moreno-Hagelsieb G, Wang Z, Walsh S, ElSherbiny A (2013). Phylogenomic clustering for selecting non-redundant genomes for comparative genomics. Bioinformatics.

[31] Moreno-Hagelsieb G. SequenceTools: getRBH.pl. 2020. https://github.com/Computational-conSequences/SequenceTools. Accessed 10 Oct 2020.

[32] Conway JR, Lex A, Gehlenborg N (2017). UpSetR: an R package for the visualization of intersecting sets and their properties. Bioinformatics.

[33] R Core Team (2020). R: A language and environment for statistical computing.

