# Supplementary material for: Progress in quickly finding orthologs as reciprocal best hits: comparing blast, last, diamond and MMseqs2
Source: BMC Genomics. 2020 Oct 24;21:741. doi: 10.1186/s12864-020-07132-6 (PMC7585182; doi:10.1186/s12864-020-07132-6)
Supplement: Supplementary file 1 — Additional file 1 Supplementary Figures. [file 12864_2020_7132_MOESM1_ESM.pdf]

Progress in quickly finding orthologs as reciprocal best hits: comparing blast, last, diamond and MMseqs2
—Supplementary Figures—

Julie Hérnandez-Salmerón and Gabriel Moreno-Hagelsieb

August 29, 2020

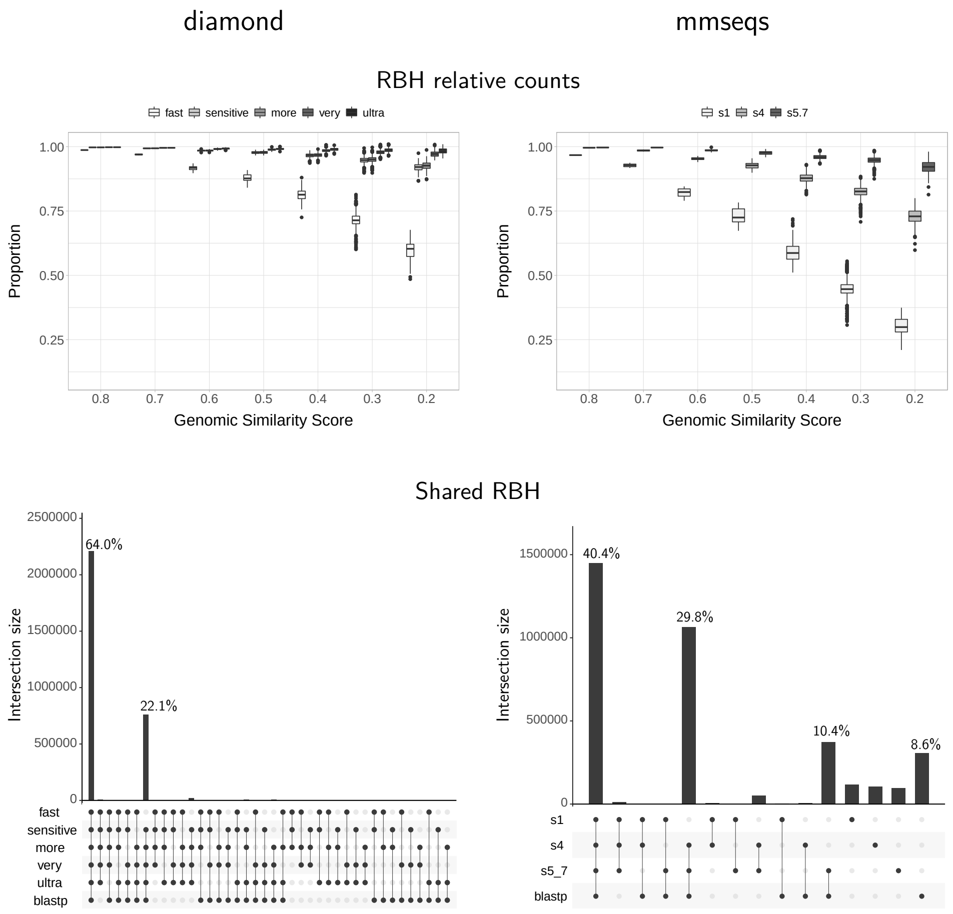

Figure S1: **Reciprocal best hits found by diamond and mmseqs with *Bacillus subtilis* as reference proteome.** The proportion of RBH found is comparable to those found by blastp when the proteomes involved are very similar (high Genomic Similarity Scores, GSS). As expected, low sensitivity options reduced the proportion of RBH found.

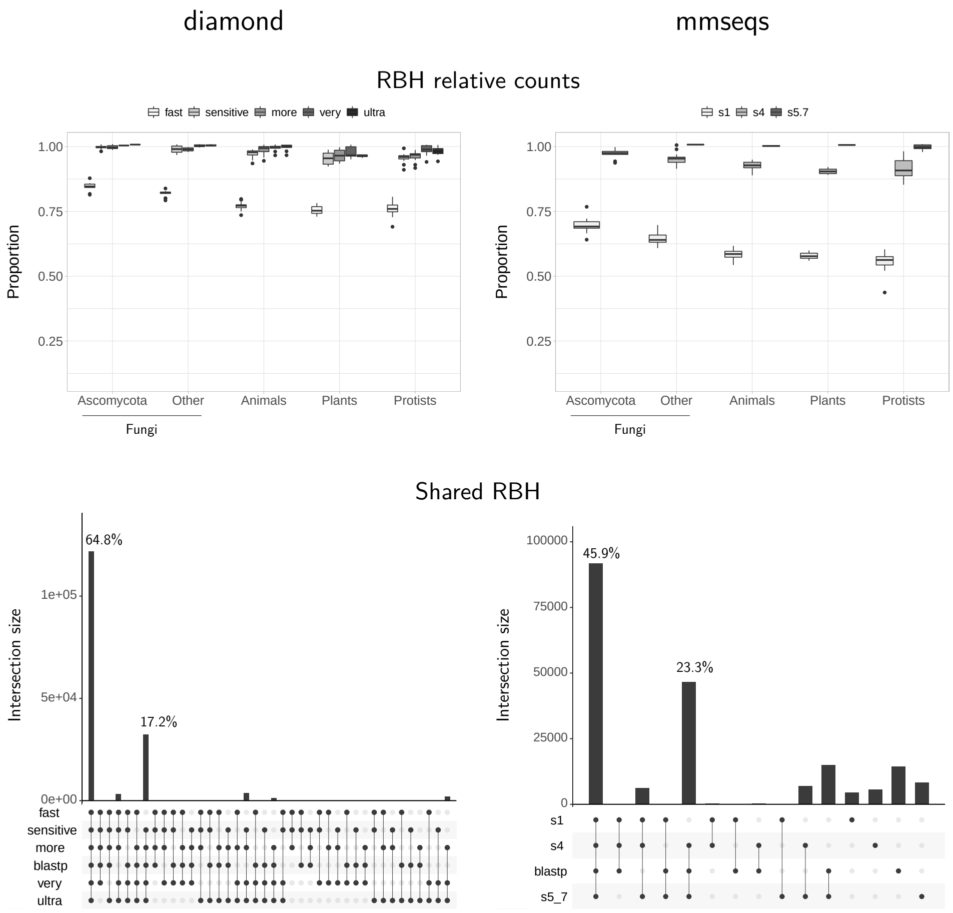

Figure S2: **Reciprocal best hits found by diamond and mmseqs with *Saccharomyces cerevisiae* as reference proteome.** The proportion of RBH found is comparable to those found by blastp when the proteomes involved are in the same taxonomic category (Ascomycota). As expected, low sensitivity options reduced the proportion of RBH found.

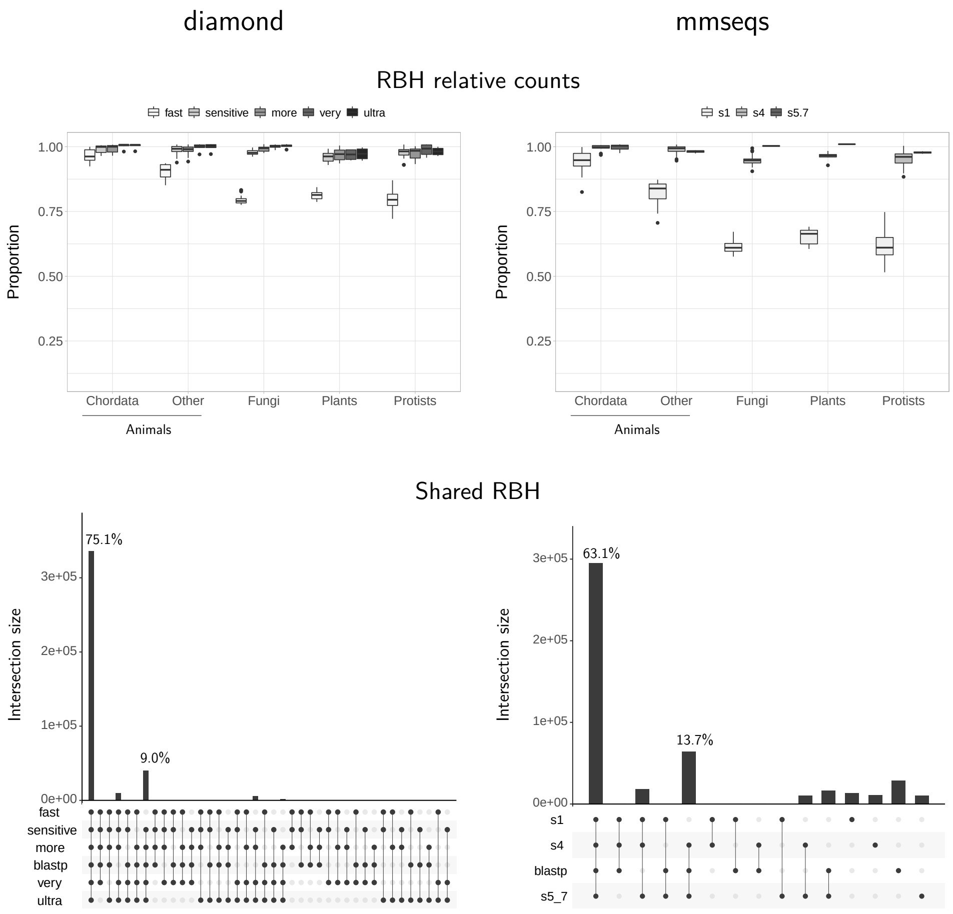

Figure S3: **Reciprocal best hits found by diamond and mmseqs with *Mus musculus* as reference proteome.** The proportion of RBH found is comparable to those found by blastp when the proteomes involved are in the same taxonomic category (Chordata). As expected, low sensitivity options reduced the proportion of RBH found.
